# Manipulation of small particles at solid liquid interface: light driven diffusioosmosis

**DOI:** 10.1038/srep36443

**Published:** 2016-11-03

**Authors:** David Feldmann, Salim R. Maduar, Mark Santer, Nino Lomadze, Olga I. Vinogradova, Svetlana Santer

**Affiliations:** 1 Institute of Physics and Astronomy, University of Potsdam, 14476 Potsdam, Germany; 2 A.N. Frumkin Institute of Physical Chemistry and Electrochemistry, Russian Academy of Sciences, 31 Leninsky Prospect, 119071 Moscow, Russia; 3 Department of Physics, M. V. Lomonosov Moscow State University, 119991 Moscow, Russia; 4 Max Planck Institute of Colloids and Interfaces, 14424 Potsdam, Germany; 5 DWI-Leibniz Institute for Interactive Materials, RWTH Aachen, Forckenbeckstraße 50, 52056 Aachen, Germany

## Abstract

The strong adhesion of sub-micron sized particles to surfaces is a nuisance, both for removing contaminating colloids from surfaces and for conscious manipulation of particles to create and test novel micro/nano-scale assemblies. The obvious idea of using detergents to ease these processes suffers from a lack of control: the action of any conventional surface-modifying agent is immediate and global. With photosensitive azobenzene containing surfactants we overcome these limitations. Such *photo-soaps* contain optical switches (azobenzene molecules), which upon illumination with light of appropriate wavelength undergo reversible *trans-cis* photo-isomerization resulting in a subsequent change of the physico-chemical molecular properties. In this work we show that when a spatial gradient in the composition of *trans-* and *cis-* isomers is created near a solid-liquid interface, a substantial hydrodynamic flow can be initiated, the spatial extent of which can be set, e.g., by the shape of a laser spot. We propose the concept of light induced diffusioosmosis driving the flow, which can remove, gather or pattern a particle assembly at a solid-liquid interface. In other words, in addition to providing a soap we implement selectivity: particles are mobilized and moved at the time of illumination, and only across the illuminated area.

In modern semiconductor industry the production of microchips must be conducted in high-level class clean rooms in order to avoid the adsorption of dust (organic or inorganic micron- to submicron sized particles) on structured surfaces. The reason is simple: small particles adsorb rather tightly to a flat surface due to strong adhesion forces. There is up to now no macroscopic brush-like device that is suitable for gentle particle removal. Conventional mechanical approaches to clean surfaces, e.g., high pressure gas or liquid flow tend to destroy functional elements along with driving the contaminants in an uncontrollable manner.

For (re-)moving or manipulating objects on a nano- and micro- meter scale there is no immediate concept such as a “miniaturized brush” that one could use to gently move a collection of objects within a selected, possibly very small area. Controlled motion of micro/nano- objects has been achieved with probing single particles at a time as with the tip of an Atomic Force Microscope (AFM)^1^,^2^,^3^. In fact, AFM is to date the only method for controlled relocation of adsorbed sub-micron sized objects, but not suitable for assemblies exceeding but a few particles.

For freely movable objects, e.g., dispersed in solution, a number of other concepts has been devised. For instance, one has made use of molecular motors as found in nature^4^,^5^,^6^,^7^; externally actuated motion employing optical tweezers^8^,^9^; optically induced dielectrophoresis^10^,^11^,^12^; and other techniques that utilize gradients of electric or magnetic fields^13^,^14^. However, all these methods cease to work as soon as the objects to be manipulated become trapped at some interface or surface, and one is essentially led back to the AFM approach.

Several years ago we have explored a different path towards moving and manipulating large ensembles of small objects. We demonstrated that particles otherwise firmly attached to a thin polymer film can be moved without explicit external contact, just by inducing local shape changes and changes in composition of an underlying polymer film, triggered by an external stimulus^15^,^16^,^17^. The particle simply follows the changing landscape in surface energy. Although the approach works well, it requires a sophisticated modification of the solid substrate that might be feasible only in a few situations.

Our work has clearly shown that in pursuing this idea further, one ideally would like to have remote, contactless control over a large ensemble of particles at arbitrary interfaces. From a general point of view, one has to solve the following problems all at once: minimizing the surface/particle interaction, generating forces locally to address only a selected set of particles, and employing a convenient remote trigger such as light. To achieve this we propose in this work the concept of a *photo-sensitive soap*. While being similar to applying a conventional soap, we shall additionally make explicit use of the functional properties of a photo-responsive unit incorporated into surfactant molecules.

The properties of many molecules can be modified with optical stimuli by incorporating a suitable photosensitive group into their structure. One of the best known molecules for this purpose is azobenzene (Fig. 1a). Azobenzene undergoes reversible photo-isomerization between a more stable *trans-* and a metastable *cis-* conformation during illumination with light of appropriate wavelength. Along with the conformational changes, the physical properties of the molecules are also altered: their shape switches from rod-like to “Γ -like” with a corresponding increase in free volume, the dipole moment increases from 0 Debye in the *trans-* to ~3 Debye in the *cis-* conformation. When incorporated into the hydrophobic tail of a surfactant molecule, photoisomerization can thus alter the hydrophobicity/hydrophilicity of the whole surfactant structure^18^, and can be toggled by alternating illumination with UV and blue light^19^. In this way, azobenzene containing surfactant bears an enormous potential for practical applications. One can, for instance, trigger compaction and decompaction of a DNA molecule by light in a reversible way^20^,^21^,^22^,^23^,^24^,^25^,^26^,^27^,^28^,^29^,^30^, or remotely control the size of microgels by applying periodically UV and blue irradiation^31^,^32^,^33^. One can modify polymer brushes with these molecules and make them photoresponsive with respect to topography and surface energy^34^,^35^,^36^. Geared towards applications in microfluidics, it was shown that a Marangoni-type flow can be induced by *cis-trans* isomerization of azobenzene solutes at the liquid-air or liquid-liquid interface; this effect has been used to steer oil droplets on an aqueous subphase^37^.

In contrast, working with particle based systems, we have discovered a peculiar phenomenon related to colloids located at a liquid-solid interface immersed into aqueous solution of photosensitive surfactant. Here we report on how small particles trapped at a solid/liquid interface can be moved by diffusioosmotic focusing initiated by the photoisomerization process. We will provide a theoretical account of how the local liquid flows emerge. It will turn out that the phenomenon is best understood as light-driven diffusioosmosis. In this way, the surfactant becomes a *photo-soap* in a new sense: not only particle-surface interaction is reduced, but also control over the “rinsing” of contaminants can be gained.

## Results

**Figure 1:**
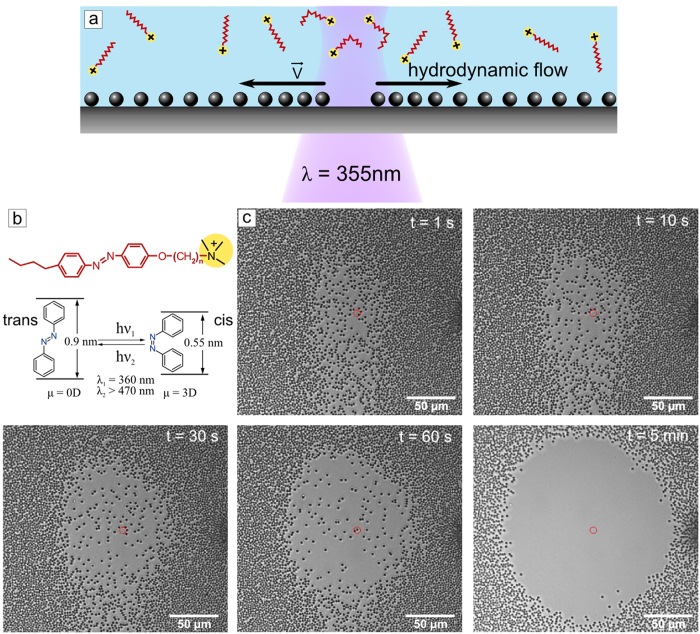
(**a**) Scheme of the experimental setup, consisting of a micro-channel filled with surfactant-water solution. Silica particles of 2 μm in diameter are adsorbed at the solid-liquid interface. Subsequently, irradiation with UV light is initiated, and a local removal of particles is achieved. (**b**) Chemical structure of an azobenzene containing cationic surfactant. Shown below is a scheme of the photo-isomerization of the azobenzene group. (**c**) Five snapshots (after irradiation time 1 s, 10 s, 30 s, 60 s and 5 minutes, documenting successive stages of the “cleansing”. A corresponding movie is provided as Supplementary Video 1.

Figure 1a shows a scheme of the experimental set-up: a glass surface with silica micro-particles (2 μm in diameter) is immersed into an aqueous solution of azobenzene containing surfactant with concentration c = 1 mM (Fig. 1b). The critical micelle concentration (CMC) of the surfactant is equal to 0.5 mM. The irradiation direction is from below, the focal plane of the UV laser (λ = 355 nm, P = 1.5 μW) is at the level of the interface where the particles are adsorbed. Before irradiation the silica particles form a densely packed monolayer at the glass surface. When illumination with UV light is turned on (the red cycle in Fig. 1c indicates the center of the laser spot), the colloids are expelled from the illuminated spot leaving behind a completely clean area after 5 minutes of irradiation. The corresponding movie is presented in Supplementary Figure 1. Along with the local irradiation, we also create a spatially inhomogeneous distribution of *trans*- and *cis-* isomers, since under UV irradiation the surfactant molecules photo-isomerize correspondingly. Without the presence of the surfactant, the particle assembly does not change, ruling out heating effects or gradients in the electro-magnetic field as a possible driving mechanism.

**Figure 2:**
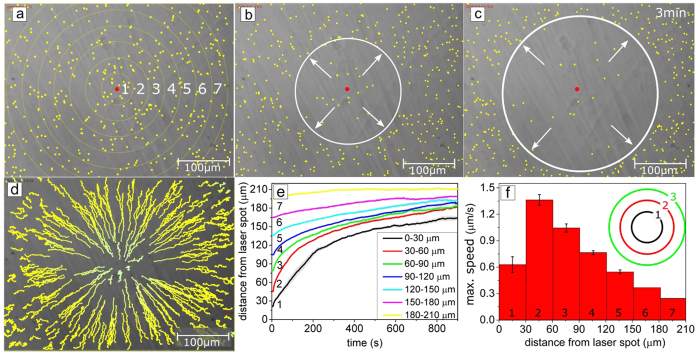
Microscope images of silica particles at the glass surface immersed in 1 mM aqueous surfactant solution before irradiation (**a**) and after irradiation during (**b**) 1 minute and (**c**) 3 minutes with UV light (λ = 355 nm, P = 1.5 μW). The particles are colorized for better contrast; the red dot depicts the center of the laser spot. The image is divided into equidistant rings around the center; the thickness of each ring element is 30 μm. (**d**) Visualization of particle trajectories. (**e**) Dependence of the particle displacement on time averaged within the different ring elements. (**f** ) Averaged maximum velocity of the particles as a function of the distance from the center of the laser spot.

We track the trajectory of each individual particle and compile corresponding statistics of particle velocities (Fig. 2).

To analyze the particle motion the micrograph was divided into ring elements of 30 μm thickness (Fig. 2a). The position of the particles enclosed in these rings was averaged and plotted as a function of time (Fig. 2e). As can be seen from Fig. 2e the particle motion differs depending on the initial position relative to the center of the laser spot. From these distance-time curves a maximal average velocity for each ring was calculated. A maximal average speed of 1.3 μm/s is obtained for particles in the ring between 30 μm and 60 μm from the center of irradiation (Fig. 2f).

The qualitative behavior of particle velocities can naturally be explained if we assume that a focused hydrodynamic flow pattern exists that drives the particles. Due to mass conservation, we expect the velocity (assuming a quasi-2D flow pattern) to drop proportionally to 1/r for increasing radial distance r from the center of irradiation. The low velocities measured directly in the vicinity of the center are then naturally explained by the necessary stagnation flow pattern there. We will come back to this point in more detail in the theoretical part presented below. We should note that particle removal is not restricted to monolayers. Also multilayers can be cleaned up locally in the very same way, and just as quickly (See Supplementary Video 2).

**Figure 3:**
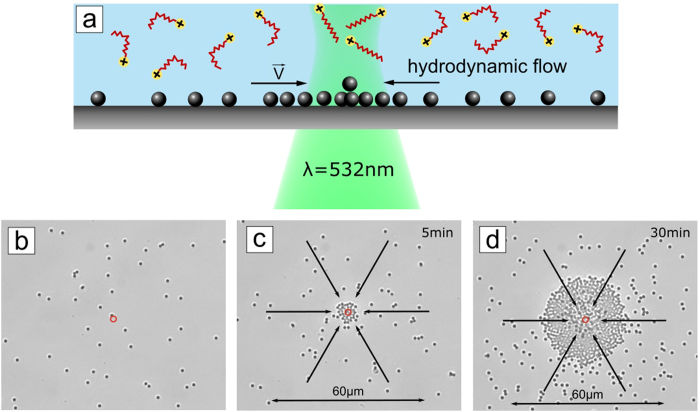
(**a**) Scheme of the inverted process. (**b**) Optical micrographs of silica particles of 2 μm diameter adsorbed on a glass surface immersed into aqueous solution with photosensitive surfactant in *cis-* conformation (c = 1 mM), obtained after global illumination with UV (10 min). (**c**–**d**) The sample is irradiated locally with green light triggering back isomerization from *cis-* to *trans-*. In this case, the particles are gathered, that is drawn towards the maximum of irradiation intensity. The direction of particle movement is indicated by black arrows. A corresponding movie is provided with the Supplementary Video 3).

As demonstrated above, under UV irradiation the particles move out of the irradiated area. In all experiments we observe a saturation effect, e.g. starting from a certain time, the particle-free area does not grow any more. The direction of particle motion can be reversed by inverting the distributions of *cis-* and *trans-* isomers. This can be achieved by first bringing the majority of the surfactants in a *cis-* state by global irradiation with UV light, and then illuminating locally with green light that triggers the return to *trans-*, see Fig. 3. In this case, during irradiation with light of λ = 532 nm, the particles move towards the center of the laser spot (Fig. 3). The gathering of colloids at the center of the laser spot continues during the whole irradiation period and results in the formation of a pile of particles (Fig. 3). Corresponding movies for particles of 2 μm and 7 μm diameter are provided as Supplementary Videos 2 and 3. Note that there is no limitation to how long this process can be run, as the supply of *cis*- isomers can be kept constant by a simultaneous global illumination with UV light. With increasing amount of gathered particles the velocity distribution is naturally affected, but within the first stages of illumination (with only few particles accumulated), the trend of the particle velocity as a function of radial distance from the laser spot center is similar to that of the outward flow pattern (induced by UV irradiation). Particles situated somewhat off the center (in our examples roughly 50 μm) show a maximal velocity of 1.3 μm/sec (Supplementary Figure 1).

**Figure 4:**
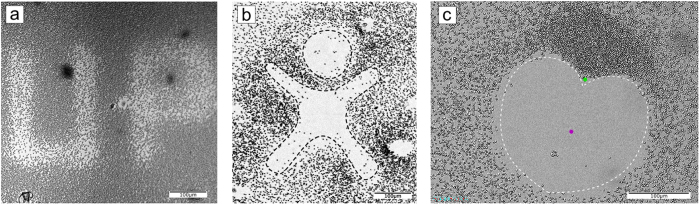
(**a**,**b**) Structuring of a particle monolayer by lateral repositioning the laser spot across the solid liquid interface: Logo of the University of Potsdam (UP) and the “happy man”. (**c**) “Heart” shaped pattern inscribed by simultaneously irradiating with UV (red dot) and green light (green dot). The corresponding video is provided as Supplementary Video 5.

Although this phenomenon could just be used for local removal (cleaning) or gathering of particles, one may also use it for structuring particle assemblies. As a demonstration, we have patterned the logo of the University of Potsdam (UP) (Fig. 4a) or a “happy man” (Fig. 4b) into the particle assembly. Figure 4c shows the result of simultaneous illumination with UV and green light at two different, spatially separated areas. The shape is the result from a combination of particle gathering and expulsion.

**Figure 5:**
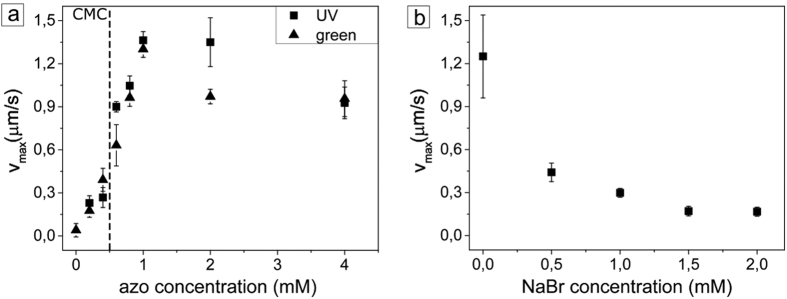
(**a**) Maximal speed of silica particles of 2 μm in diameter as a function of surfactant concentration for two different irradiation wave lengths: UV (squares), and green light (triangles). The CMC of the surfactant concentration is designated by the dashed red line. (**b**) Maximal velocities for UV irradiation as a function of salt (NaBr) concentration.

As far as we know, the maximal velocity of particles is not affected by particle size, at least down to 1 μm and as large as 40 μm (see Supplementary Figure 2). But there is a strong dependence of the particle velocity on the surfactant concentration. Indeed, Fig. 5a shows the maximal radial velocity of the particles at different surfactant concentrations for two different directions of motion: outwards (induced by UV irradiation, λ = 355 nm) and inwards (during irradiation with green light, λ = 532 nm).

The velocity of the particles increases with azobenzene concentration peaking at roughly c = 1 mM. The behavior of v_max_ is similar for both, the inwards and outwards motion of the particles, under UV or green irradiation, respectively (Fig. 5a). Another parameter affecting the particle velocity is the ionic strength of the surfactant solution. With increasing salt concentration the particle speed decreases as shown in Fig. 5b.

**Figure 6:**
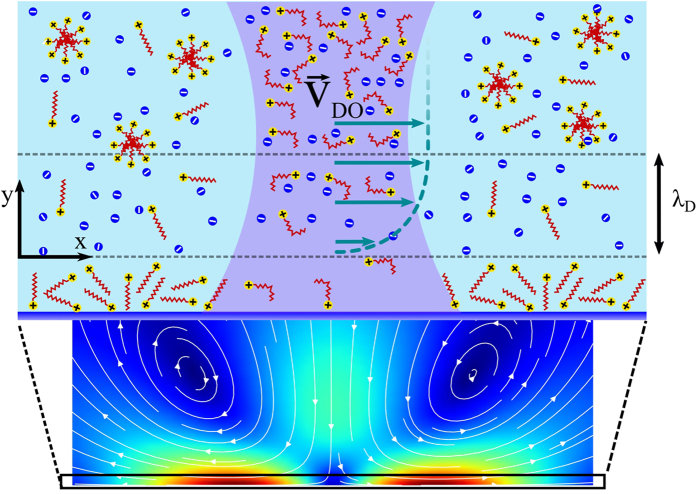
Top: schematic molecular scale view of light-driven generation of local diffusioosmotic (DO) flow inside and outside the EDL. The diffusioosmotic velocity, *v*_*DO*_, is defined at a distance *λ*_*D*_ away from the solid surface. Bottom: schematic representation of streamlines of bulk liquid flow (far from the surface). The extent of the upper schematic region is indicated by the black solid rectangle. The velocities are calculated numerically using Stokes’ equations with *v*_*DO*_ as input parameter obtained from experiment (presented in Fig. 2).

Summarizing all experimental results, we suggest the following general picture of how particle motion is governed. Let us first consider the case of UV illumination. As can be seen in Fig. 6, during focused irradiation with UV light, a corresponding local excess of *cis* isomers is created. This results in a gradient of relative *cis-* and *trans-* isomer concentrations.

First, the light intensity distribution within the laser spot is inhomogeneous and in this way naturally generates a gradient of concentration of different isomers; second, newly created *cis-*isomers will diffuse out of the center of irradiation, modifying the laser induced distribution. At the solid-liquid interface this can in principle lead to a gradient of concentration of adsorbed surfactant, but this could not generate any classical Marangoni flow since adsorbed molecules are immobile. An explanation for emerging liquid flow can be obtained if we invoke diffusioosmosis, which originates due to gradients of *cis-* and *trans*- isomer concentrations in the lateral direction (Fig. 6) and is driven by an electrostatic diffuse layer (EDL) of thickness λ_*D*_, i.e., the region where the surface charge is balanced by the cloud of counter-ions^38^,^39^,^40^,^41^,^42^.

Indeed, the spatially varying excess concentration of counter-ions near the solid leads to a corresponding osmotic pressure:


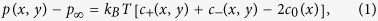


where *c*_0_(*x*) is the bulk concentration of surfactant molecules, 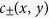 local concentrations of ions within the EDL, *k*_*B*_ is the Boltzmann constant, and *T* is the temperature. The lateral gradient in *cis-* concentration induced during illumination (along the *x* direction in Fig. 6) then causes an osmotic pressure gradient driving a hydrodynamic flow. The flow field ν satisfies Stokes’ equations:


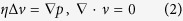


By solving Eq. (2) in the inner region, i.e. within EDL, we can obtain the x-component of diffusioosmotic velocity at the EDL plane, 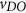 (Fig. 6):


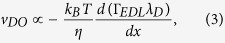


with the EDL surface excess of ions given by


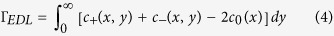


Eq. (3) is similar to derived earlier^43^, but here 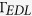 depends on *x*. We recall that the addition of NaBr leads to a decrease in fluid velocity (see Fig. 5b), which is in agreement with our model.

The gradient of surface excess in Eq. (3) can be expressed in terms of concentration of either *trans-* or *cis*-isomer generated by local irradiation:


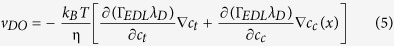


where indices *t* and *c* correspond to trans- and cis- isomers. The quantities 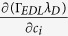 characterize the adsorption of *trans-* and *cis-* molecules (in thin layer near solid/liquid interface, Fig. 6) and can be estimated experimentally by measuring the surfactant adsorption isotherm and surface zeta potential. Note that we can relate 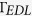 to electrostatic surface potential, *ϕ*_*0*_, by employing the Boltzmann distribution of ions inside the EDL, which together with Grahame’s equation leads to:^44^,^45^


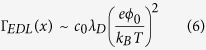


where we assume *ϕ*_*0*_ does not exceed 100 mV. It follows that the flow depends strongly on total surfactant concentration 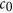 and on the adsorption strength of *cis-* and *trans-* isomers, which alters the surface zeta potential. We remark that this inner flow generates an outer flow shown on Fig. 6, and we refer the reader to Supplementary Information (Figure S3) for computational details.

We shall now focus on difference between situations below and above the CMC. Below the CMC *c*_0_ < *c*_cmc_, lower velocity is related to two types of opposite gradient of *cis-* and *trans-* molecules. Since below CMC *c*_*c*_ = *c*_0_ − *c*_*t*_, we obtain


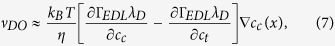


so that the velocities’ magnitude and sign depend on the difference of adsorption constants for *cis* and *trans* isomers. Above the CMC, the concentration of *trans* molecules stays nearly constant, 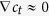 due to micelle formation, and only 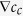 is important, so we derive:


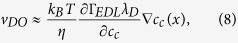


which suggests that an increase in velocity occurs for concentrations *c*_0_ > *c*_*cmc*_. In this case the concentration of single molecules is no longer described by *c*_*c*_ = *c*_0_ − *c*_*t*_ due to micelle decomposition. Let us now estimate the diffusioosmotic velocity by using the above arguments:


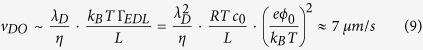


**Figure 7:**
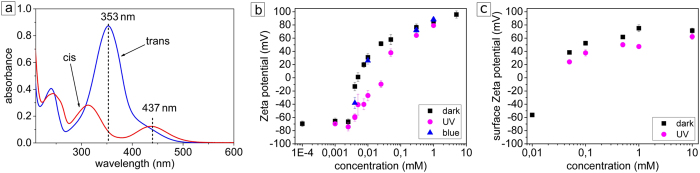
(**a**) UV absorption spectra of *trans* (blue) and *cis* (red) isomers of azobenzene surfactant. (**b**) Dependence of the particle and (**c**) glass surface zeta potential on the surfactant concentration.

Here the diameter of the laser spot, L, is 60 μm, *λ*_*D*_ ~ 10 nm at c_0_ = 1 mM, and η ~ 1 mPa^·^s, and the value of *ϕ*_*0*_ = 70 mV is deduced from the experimental data on the zeta potential, see Fig. 7c. The value of 7 μm/s is of the order of the measured maximal velocity validating our model. We note that the magnitude of velocities is much lower than those for typical Marangoni flows found at liquid air interfaces^46^,^47^. This is mainly because 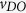 scales with the screening length *λ*_*D,*_ but not with the film thickness as in the case of Marangoni flow.

In summary, we have shown that with the local gradients imposed on the distribution of *cis-* and *trans-* isomers close to a solid-liquid interface, sufficiently strong hydrodynamic forces can be generated in order to explain the observed velocity distributions of a particle assembly at the solid substrate. We should recall that the photo-soap is still acting in the conventional way, that is, it reduces the surface-particle interactions and renders the particles mobile.

## Conclusions

We have demonstrated how a photosensitive surfactant was employed to generate sufficiently strong local hydrodynamic forces to move and pattern particle assemblies at a solid liquid interface. This *photo-soap* is to serve two purposes simultaneously: modification of interfacial energies and removing of particles by initiation of local hydrodynamic shear flow. Particles are moved at the time of illumination, and only within and in the vicinity of the illuminated area. Whether a flow in or out of the illuminated area occurs is governed by the wavelength employed: upon UV irradiation promoting *trans-cis* isomerization we observe outflow, whereas employing green light inducing back-isomerization from *cis* to *trans* inflow can be generated, which causes particles to pile up at the stagnation point at the center of the flow pattern.

Furthermore, moving a single laser spot across a dense particle layer can be used for straightforward spatial patterning; more complex operations are possible by employing several spots simultaneously.

We have shown that the resulting particle velocities do not depend on particle diameter (at least in the range of 1 μm to 10 μm), but are strongly impacted by surfactant concentration. The largest velocities are observed above the CMC, where micelles form effective reservoirs setting free a large amount of surfactant molecules at once upon irradiation. Our theoretical analysis of the phenomena described suggests that the initiation of flow can be understood with the concept of light driven diffusioosmosis. Here the spatial gradients in both, *cis* and *trans* isomers generate corresponding gradients in osmotic pressure that in the presence of the solid substrate leads to an effective shear flow. From a practical point of view, we hope that our approach of using a photosensitive soap can be turned into an application that alleviates some of the problems encountered in cleaning processes or in manipulating assemblies of adsorbed sub-micron sized particles.

## Experimental Section

### Materials

Azobenzene containing cationic surfactant with a spacer of six methylene groups between the positively charged trimethylammonium bromide head group and the azobenzene unit was synthesized as described elsewhere^48^. The surfactant was dissolved in Milli-Q water and diluted to the required concentrations.

The photoisomerization behavior of the surfactant is described in detail elsewhere^22^. In short, in the dark state (*trans* conformation) of the surfactant is characterized by an absorption band with a maximum at 353 nm, while the *cis* isomer is characterized by two absorption bands with maxima at 313 nm and at 437 nm (Fig. 7a). The band with maximum at ~240 nm presented in both isomers corresponds to the absorption of the π-conjugated benzene rings. The lifetime of the *cis* isomer in the dark is 48 hours, while the photo-isomerization from cis- to trans-state under irradiation with green light (λ = 532 nm, P = 30 μW) takes place within a few seconds, approaching a photo-stationary state after 10 minutes of irradiation.

The surfactant solution was introduced into a flow chamber (μ-Slide VI 0.4 flow chamber, Ibidi, Germany) with a volume of 60 μl. Aqueous solution of silica particles of varying sizes (Micromod, Germany) was added to and mixed with the surfactant solution. To prevent evaporation, the chamber openings were sealed with Parafilm (Bemis Company, Inc., USA). Before measurement the samples are kept in dark for several minutes until the silica particles sediment to the bottom of the substrate.

### Methods

#### Zeta potential

The zeta potential of silica particles was measured using a Zetasizer (Nano-ZS, Malvern Instruments Ltd.). The azobenzene concentration was varied keeping the particle concentration fixed at 0.04 mg/ml. In water solution the silica particles are negatively charged with a zeta potential of −65 mV. With increasing surfactant concentration, the zeta potential rises up to a value of ca. +80 mV for c = 1 mM, irrespective of whether the surfactant is in *trans-* or *cis-* conformation (Fig. 7b). At concentrations starting from 0.1 mM the silica particles are positively charged for both, *trans-* and *cis*-isomers.

The surface zeta potential of a glass surface was measured using a surface zeta potential cell ZEN1020 (Malvern Instruments Ltd, UK). Azobenzene surfactant was dissolved in water and diluted to a certain concentration and mixed with polystyrene nano spheres (Thermo Fisher Scientific Inc. USA) so that the suspension was slightly milky. About 1.2 ml was filled into a plastic cuvette (DTS0012, Malvern Instruments Ltd, UK). A glass substrate was cut and broken to a width of ca. 4 mm to fit the size requirements of the cell.

#### Microscopy

An inverted Olympus IX71 was equipped with two lasers (Samba 532 nm, Cobolt, Sweden; 355 nm, Genesis CX, Coherent Inc., USA). The laser beams were focused through the objective of the microscope to the solid-liquid interface where the particles adsorbed. The laser power was measured at the solid/liquid interface by optical power meter 1918-R with sensor 918D-UV-OD3R (Newport Corporation,Irvine, CA, USA). Additionally, the setup was kept in the dark to prevent uncontrolled isomerization. Images were acquired with an Olympus XM10 monochrome camera at a speed of 1 frame per second.

#### Tracking and data analysis

Particles are tracked using the Mosaic Single Particle Tracking plugin for ImageJ (Rasband, W.S., ImageJ, U. S. National Institutes of Health, Bethesda, Maryland, USA, http://imagej.nih.gov/ij/, 1997–2015). The tracking algorithm was described by Koumoutsakos^49^. The particle tracks were analyzed using a self-written Matlab script: for each particle and frame the distance from the laser spot is calculated. The area around the spot is divided into ring segments of defined thickness of 30 μm. For each particle, the distance at t = 0 is calculated and averaged over all particles in one ring segment. In addition, the velocities are calculated by dividing the distance moved by the time interval Δt = 1 s. Maximal velocities are calculated with a time difference of Δt = 10 s smoothing the velocity profile.

## Additional Information

**How to cite this article**: Feldmann, D. *et al.* Manipulation of small particles at solid liquid interface: light driven diffusioosmosis. *Sci. Rep.* **6**, 36443; doi: 10.1038/srep36443 (2016).

**Publisher’s note:** Springer Nature remains neutral with regard to jurisdictional claims in published maps and institutional affiliations.

## Supplementary Material

Supplementary Information

Supplementary Video 1

Supplementary Video 2

Supplementary Video 3

Supplementary Video 4

Supplementary Video 5
