# Supplementary material for: Manipulation of small particles at solid liquid interface: light driven diffusioosmosis
Source: Sci Rep. 2016 Nov 3;6:36443. doi: 10.1038/srep36443 (PMC5093767; doi:10.1038/srep36443)
Supplement: Supplementary Information [file srep36443-s1.doc]

**Supplementary Information**

**Manipulation of small particles at solid liquid interface: light driven diffusioosmosis**

David Feldmann,^1^ Salim R. Maduar, ^2,3^ Mark Santer, ^5^ Nino Lomadze, ^1^  Olga I. Vinogradova, ^2,3,4^ Svetlana Santer^1^

*^1^ Institute of Physics and Astronomy, University of Potsdam, 14476 Potsdam, Germany*

*^2^ A.N. Frumkin Institute of Physical Chemistry and Electrochemistry, Russian Academy of Sciences,*

*31 Leninsky Prospect, 119071 Moscow, Russia*

*^3^ Department of Physics, M. V. Lomonosov Moscow State University, 119991 Moscow, Russia*

*^4^ DWI-Leibniz Institute for Interactive Materials, RWTH Aachen, Forckenbeckstraße 50, 52056 Aachen, Germany*

*^5^ Max Planck Institute of Colloids and Interfaces, 14424 Potsdam, Germany*

**Supplementary Video 1.** Video of the particle (d=2μm) motion during irradiation with UV light (λ=355nm, P=1.5µW). The particles are adsorbed on a glass surface and immersed in an aqueous solution of photosensitive surfactant of concentration c=1mM.

**Supplementary Video 2.** Video of the removal of the multilayer of silica particles (d=2mm) during irradiation with UV light (λ=355nm, P=1.5µW).

**Supplementary Video 3.** Video of the particle (silica particle of d=2μm) motion during irradiation with green light (λ=532nm, P=30µW). The particles are gathered to the center of the laser spot.

**Supplementary Video 4**. Video of the motion of the silica particles of 7μm in diameter under irradiation with light of λ=532nm (P=30µW).

**Supplementary Video 5**. Video of the motion of silica particle (2μm) under simultaneous irradiation with UV (red dot) and green light (green dot).


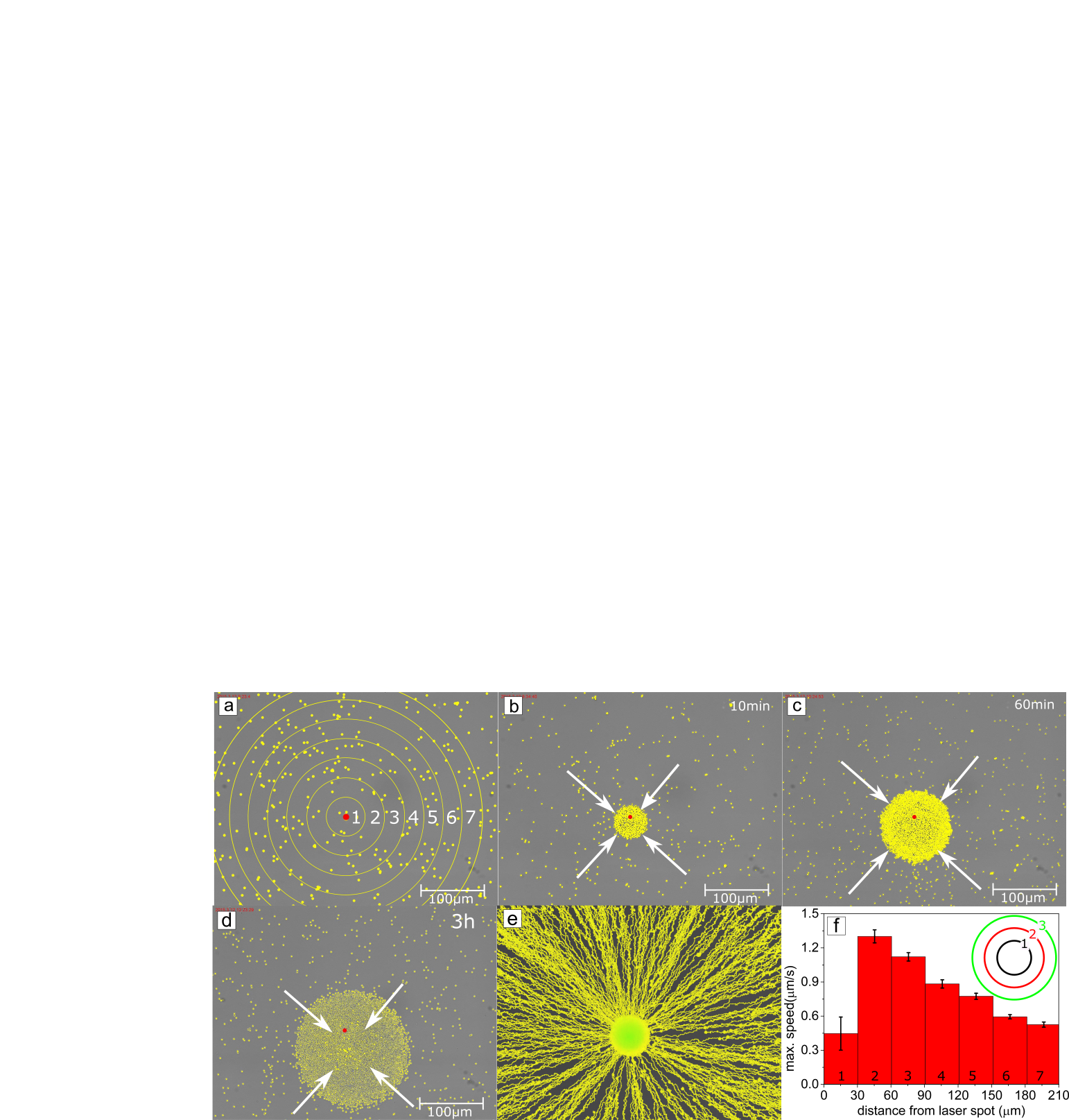


**Supplementary Figure 1.** (a) Before irradiation, the particles are randomly distributed on a glass surface. Immediately after turning on the irradiation with green laser (λ=532nm), particles start to move towards the center (marked by red point) of irradiated area (b-d). Images are divided into equidistant rings of thickness 30μm around the laser spot marked by red point. The motion proceeds over the irradiation, here shown during 3 hours. The traces of the gathered particles are shown in (e). (f) Velocity distribution as a function of the particle position within the laser spot. The position is indicated by the rings number as shown in the insert.


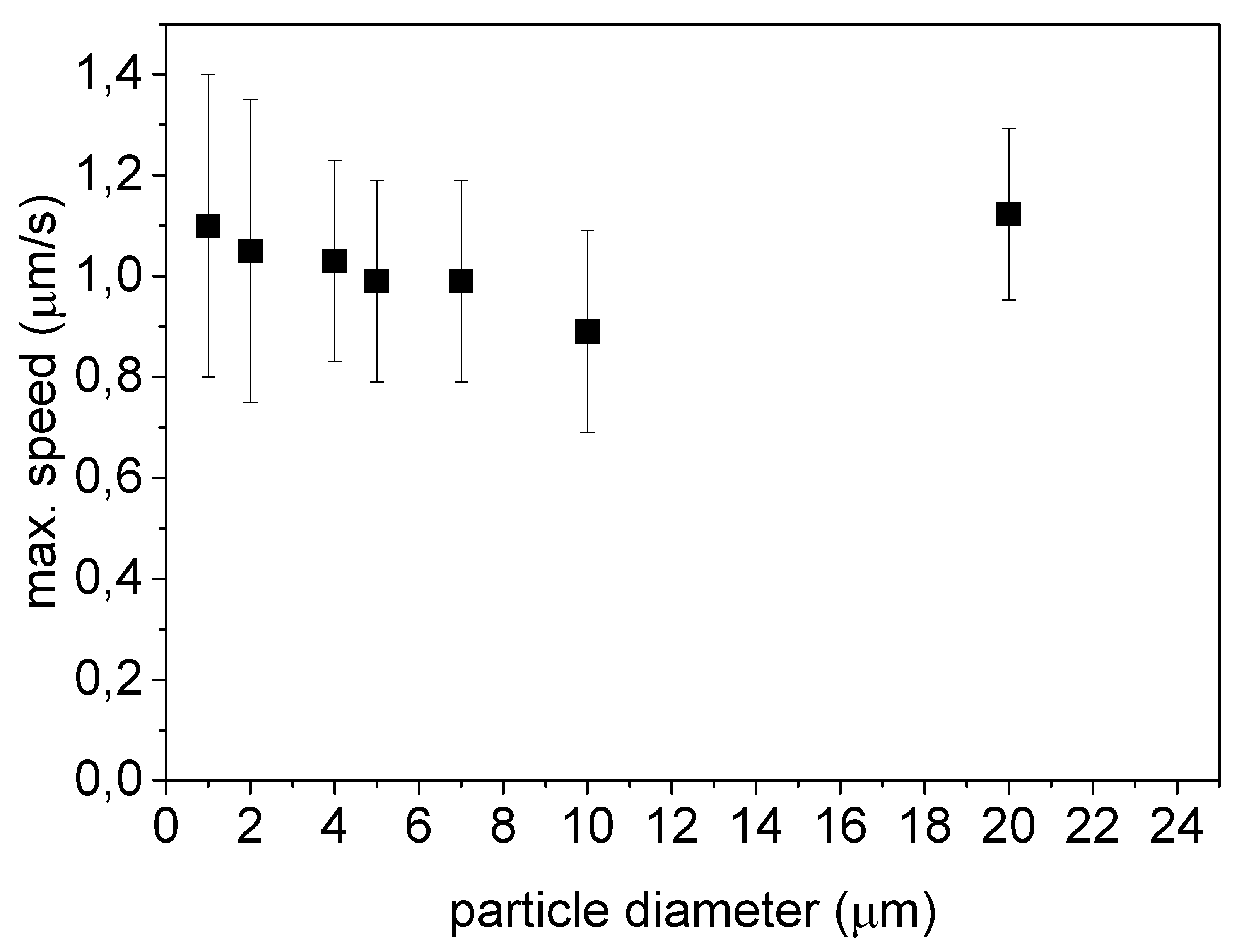


**Supplementary Figure 2.** Dependence of the maximum velocity of silica particles immersed in an equation solution of the surfactant (c=1mM) on the diameter.

**Supplementary Figure 3.** Numerical calculations of the outer (bulk) velocity profile.

Bulk flow profile may be calculated by using Eq.(2) of the main text. We can eliminate the pressure term from this equation by applying the operator $\nabla\times$:

(1)

We then introduce a stream function that satisfies a continuity equation defined in Eq.(2) of the main text:

and (2)

The boundary conditions can be written as:

(3)

(4)

We remark that to calculate an outer flow profile as first boundary condition we use *v_DO_* defined in the main text, which is a tangential velocity at the EDL plane. The second boundary condition reflects impermeability of a surface. Eq.(1) can now be rewritten as a biharmonic equation for a stream-function $\psi(x,y)$:

(5)

Outer flow profiles have been calculated numerically by using general solutions of the biharmonic equation [1] and *v_DO_(x)* deduced from the experimental data shown in Fig.2.

[1] Polyanin, A. D., Handbook of Linear Partial Differential Equations for Engineers and Scientists, Chapman & Hall/CRC, 2002."
